# Discordance in 21-gene recurrence scores between paired breast cancer samples is inversely associated with patient age

**DOI:** 10.1186/s13058-020-01327-1

**Published:** 2020-08-18

**Authors:** Sarah M. Bernhardt, Pallave Dasari, Joseph Wrin, Wendy Raymond, Suzanne Edwards, David Walsh, Amanda R. Townsend, Timothy J. Price, Wendy V. Ingman

**Affiliations:** 1 Discipline of Surgery, Adelaide Medical School, University of Adelaide, The Queen Elizabeth Hospital DX465702, 28 Woodville Rd, Woodville, Adelaide, 5011 Australia; 2 Robinson Research Institute, University of Adelaide, Adelaide, Australia; 3 Flinders Medical Centre, Flinders University of South Australia and Clinpath Laboratories, Bedford Park, Australia; 4 School of Public Health, University of Adelaide, Adelaide, Australia; 5 Adelaide Medical School, University of Adelaide, Adelaide, Australia; 6 Department of Medical Oncology, The Queen Elizabeth Hospital, Adelaide, SA Australia

**Keywords:** Premenopausal breast cancer, Predictive biomarkers, Age, Menstrual cycle, Genomics

## Abstract

**Background:**

The Oncotype DX 21-gene Recurrence Score is a genomic-based algorithm that guides adjuvant chemotherapy treatment decisions for women with early-stage, oestrogen receptor (ER)-positive breast cancer. However, there are age-related differences in chemotherapy benefit for women with intermediate Oncotype DX Recurrence Scores that are not well understood. Menstrual cycling in younger women is associated with hormonal fluctuations that might affect the expression of genomic predictive biomarkers and alter Recurrence Scores. Here, we use paired human breast cancer samples to demonstrate that the clinically employed Oncotype DX algorithm is critically affected by patient age.

**Methods:**

RNA was extracted from 25 pairs of formalin-fixed paraffin-embedded, invasive ER-positive breast cancer samples that had been collected approximately 2 weeks apart. A 21-gene signature analogous to the Oncotype DX platform was assessed through quantitative real-time PCR, and experimental recurrence scores were calculated using the Oncotype DX algorithm.

**Results:**

There was a significant inverse association between patient age and discordance in the recurrence score. For every 1-year decrease in age, discordance in recurrence scores between paired samples increased by 0.08 units (95% CI − 0.14, − 0.01; *p* = 0.017). Discordance in recurrence scores for women under the age of 50 was driven primarily by proliferation- and HER2-associated genes.

**Conclusion:**

The Oncotype DX 21-gene Recurrence Score algorithm is critically affected by patient age. These findings emphasise the need for the consideration of patient age, particularly for women younger than 50, in the development and application of genomic-based algorithms for breast cancer care.

## Introduction

Emerging in the clinic are new assays that utilise gene-expression profiling to provide an intrinsic, molecular portrait of an individual breast cancer. The expression of a panel of biomarkers is quantified in a tumour sample, and these are combined in an algorithm to predict the risk of disease recurrence and treatment response. This genomic approach promises improved treatment decision-making capabilities compared to traditional protein-based methods and is part of a new era of genomic-based precision medicine [1, 2]. Nevertheless, caution in the adoption of algorithms to guide health decisions is required. Bias against under-represented groups can occur if not explicitly accounted for, and utility of the assay may not be directly transferable to patient groups not included in its development [3, 4].

A leading genomic biomarker assay, Oncotype DX, quantifies a panel of 21 genes and combines them into an algorithm to produce a Recurrence Score that predicts the likely benefit of the addition of chemotherapy to endocrine treatment [5–10]. The Oncotype DX assay is recommended in international guidelines to guide adjuvant chemotherapy treatment decisions for women with early-stage, oestrogen receptor (ER)-positive, HER2-negative breast cancer [11–14]. The Oncotype DX assay is available to both premenopausal and postmenopausal women to assist treatment decision-making, and use of the assay results in a net reduction in chemotherapy use [15]. However, Oncotype DX was developed and validated predominantly in postmenopausal women [16], and age-related differences in chemotherapy benefit have been identified [10].

The TAILORx study [10] incorporated data from 9719 women with breast cancer, reporting that for women over the age of 50 years with Recurrence Scores less than 26 (*n* = 4495), endocrine therapy alone was not inferior to chemo-endocrine therapy in terms of disease-free and overall survival. However, women under the age of 50 with Recurrence Scores between 16 and 25 (*n* = 2216) still derived some benefit from chemotherapy. When clinical information was integrated with Oncotype DX Recurrence Scores, the prediction of which premenopausal patients would receive a substantial benefit from chemotherapy was not improved [17]. The biological basis of this age-related difference in chemotherapy benefit for women with intermediate Recurrence Scores is not well defined.

In premenopausal women, hormone receptor protein expression fluctuates throughout the menstrual cycle in response to fluctuating concentrations of oestrogen and progesterone and this is associated with downstream changes in the expression of a number of genes which are part of the Oncotype DX signature including *PGR*, *MKI67*, *CCNB1*, *BIRC5* and *MYBL2* [18–23]. Therefore, changes in gene expression with menstrual cycle stage could affect Oncotype DX Recurrence Scores. Indeed, an in vitro study suggests that the co-treatment of breast cancer cell lines with oestrogen and progesterone increases Oncotype DX Recurrence Scores, compared to oestrogen treatment alone [24].

In this study, we propose that menstrual cycling in premenopausal women affects the Oncotype DX 21-gene algorithm. We conducted a retrospective study on paired human ER-positive breast cancer samples (the biopsy and definitive surgery of the tumour) to investigate how patient age affects variability in a 21-gene experimental recurrence score, analogous to the Oncotype DX Recurrence Score, within the same tumour. If menstrual cycling affects Oncotype DX Recurrence Scores, it is hypothesised there would be increased discordance in recurrence scores between paired samples collected from younger women compared to non-cycling older women.

## Methods

### Human breast cancer sample collection

Ethics approval was obtained from The Queen Elizabeth Hospital Human Research Ethics Committee (approval number Q20170106), and informed consent was obtained from study participants. Paired formalin-fixed paraffin-embedded (FFPE) breast cancer samples were retrospectively collected from women diagnosed with invasive, ER-positive breast cancer. Patients presenting with benign breast disease or who had received any neoadjuvant therapy were excluded from the study.

**Fig. 1:**
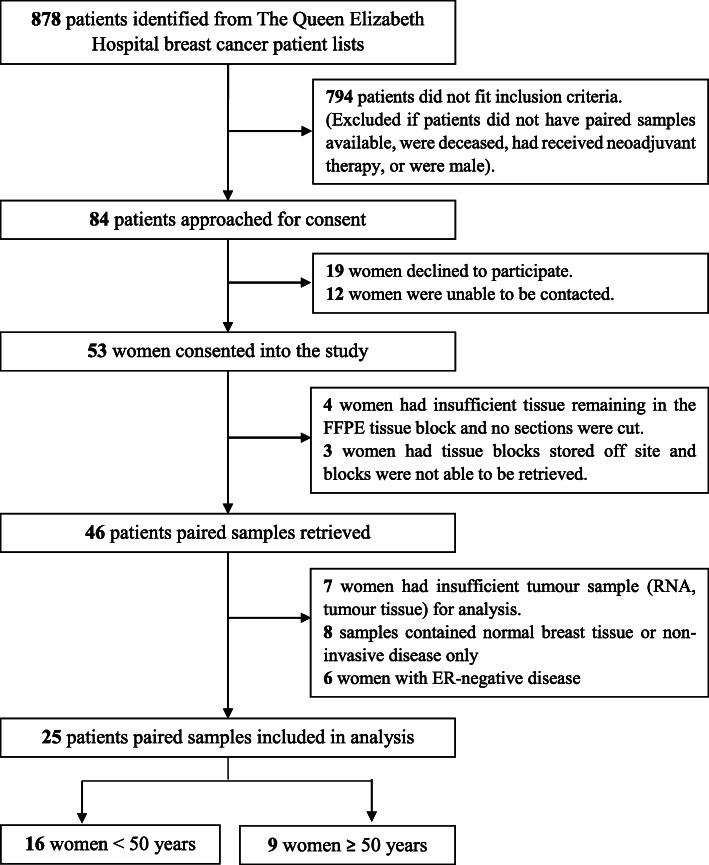
Flow chart showing patient recruitment. Potential patients for inclusion in the study were identified from The Queen Elizabeth Hospital breast cancer patient lists. Of the 878 patients initially identified, 25 women were included in the study; 16 women aged under 50 and 9 women aged over 50

Patients were identified from a list of 878 breast cancer patients who had been referred to The Queen Elizabeth Hospital Oncology Unit between 2000 and 2015. Patients were ineligible if they did not have two samples of the same tumour (i.e. the biopsy and definitive surgery of the tumour) available, were deceased, had received neoadjuvant therapy or were male. Following review for eligibility, consent was obtained and FFPE blocks were retrieved and then assessed by haematoxylin and eosin staining to confirm the presence of invasive breast cancer in each sample. All samples that met these criteria were used in the final analysis, with 25 pairs of breast cancer samples meeting these criteria. The process for identifying and recruiting patients is highlighted in Fig. 1.

### Haematoxylin and eosin staining

**Fig. 2:**
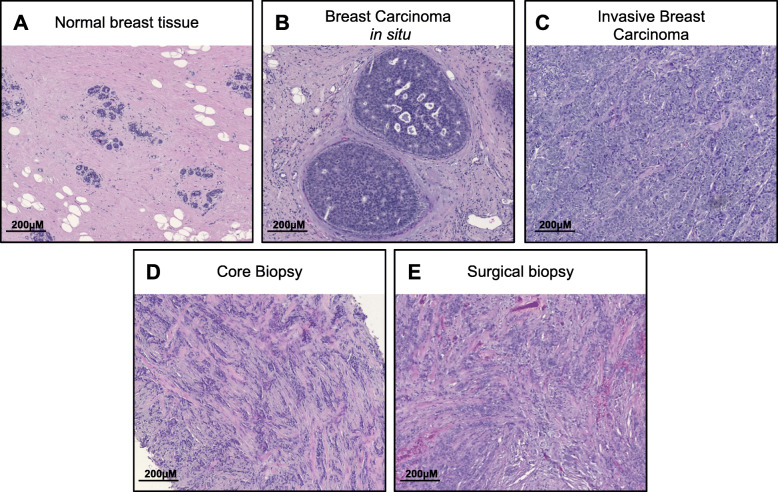
Haematoxylin and eosin stains of formalin-fixed paraffin-embedded tissue sections collected from breast cancer patients. FFPE tissue blocks were retrieved from 46 patients, and haematoxylin and eosin stains were performed to confirm the presence of malignant breast disease. Examples of **a** normal breast tissue, **b** carcinoma in situ and **c** invasive carcinoma. Samples that did not contain invasive disease were excluded from analysis. **d**, **e** Haematoxylin and eosin stain of a core biopsy and corresponding surgically excised tumour from a patient with a grade 3 invasive ductal carcinoma. Bars represent 200 μM

To confirm the presence of invasive breast cancer in the tissue sample, haematoxylin and eosin (H&E) staining was performed on 5-μM paraffin-embedded sections. Sections were dewaxed in xylene and subsequently passed through 100%, 90%, 70% and 50% ethanol for rehydration. Slides were stained with haematoxylin (Sigma-Aldrich) and counterstained with eosin (Sigma-Aldrich), prior to dehydrating and mounting with Entellan mounting medium. H&E-stained FFPE breast cancer samples were assessed by a pathologist to confirm the presence of malignant disease prior to RNA extraction. Examples of haematoxylin- and eosin-stained tissue sections are presented in Fig. 2. Other characteristics of the tumour were obtained from pathological reports completed at the time of diagnosis.

### RNA extraction and complementary DNA (cDNA) synthesis

Total RNA was extracted from 6 × 10 μm thick FFPE breast cancer tissue sections using the PureLink FFPE RNA Isolation kit (Invitrogen). cDNA was reverse transcribed from approximately 250 ng of RNA using SuperScript IV VILO with ezDNase Enzyme (Invitrogen) as per the manufacturer’s instructions.

### Quantitative real-time PCR

Custom designed 384-well TaqMan array cards (ThermoFisher) were used to measure gene expression in FFPE breast cancer samples. Primer sequences were designed by ThermoFisher. Array cards were loaded with 100 μL of 1:1 mix of cDNA and TaqMan Fast Advanced Master Mix (Applied Biosystems) and run using a QuantStudio12K Real-Time PCR system (Applied Biosystems), as per the manufacturer’s instructions. The expression of each gene was measured in duplicate.

### Calculation of 21-gene experimental recurrence scores

Normalised gene expression measurements were calculated as Δ CT = CT (mean of five reference genes) – CT (gene of interest) + 10. A 1-unit increase in reference-normalised expression measurements reflects a doubling of RNA. Experimental recurrence scores were calculated from reference-normalised gene expression, using the Oncotype DX 21-gene Recurrence Score algorithm, as described below.

To calculate experimental recurrence scores, 21-gene group scores were first calculated using normalised gene expression measurements:

$$ {\displaystyle \begin{array}{c} HER2\ \mathrm{group}\ \mathrm{score}=0.9\times GRB7+0.1\times ERBB2\\ {} ER\ \mathrm{group}\ \mathrm{score}=\left(0.8\times ESR1+1.2\times PGR+ BCL2+ SCUBE2\right)\div 4\\ {}\begin{array}{c}\mathrm{Proliferation}\ \mathrm{group}\ \mathrm{score}=\left( BIRC5+ KI67+ MYBL2+ CCNB1\right)\div 4\\ {}\mathrm{Invasion}\ \mathrm{group}\ \mathrm{score}=\left( CTSL2+ MMP11\right)\div 2\end{array}\end{array}} $$

The unscaled experimental recurrence score (RSu) was calculated from the above group scores:

$$ \mathrm{RSu}=+0.47\times HER2\ \mathrm{group}\ \mathrm{score}-0.34\times ER\ \mathrm{group}\ \mathrm{score}+1.04\times \mathrm{proliferation}\ \mathrm{group}\ \mathrm{score}+0.10\times \mathrm{invasion}\ \mathrm{group}\ \mathrm{score}+0.05\times CD68-0.08\times GSTM1-0.07\times BAG1 $$

The scaled experimental recurrence score (RS) was then calculated from the unscaled recurrence score:

$$ {\displaystyle \begin{array}{c}\mathrm{RS}=0\ \mathrm{if}\ \mathrm{RS}\mathrm{u}<0\\ {}\mathrm{RS}=20\times \left(\mathrm{RSu}-6.7\right)\ \mathrm{if}\ 0\le \mathrm{RSu}\le 100\\ {}\mathrm{RS}=100\ \mathrm{if}\ \mathrm{RS}\mathrm{u}>100\end{array}} $$

## Results

### Patient characteristics

Twenty-five patients with ER-positive, invasive breast cancer were included in the analysis. Hormone receptor status was obtained from pathology reports completed during the routine breast cancer diagnosis. Patient and tumour characteristics are detailed in Table 1. Paired samples were collected from the same tumour at different times; twenty-two women had paired core needle biopsies and corresponding surgical excisions, while three women had paired surgical excisions and corresponding re-excisions with residual disease. Sample 1 was defined as the earlier collected sample, with sample 2 as the corresponding later collected pair. The dates of sample collection were obtained from electronic medical records. Paired samples were collected an average of 18 days apart, and in the absence of any intervention.

**Table 1:** Patient characteristics

Characteristics	***n*** (%)
**Total number**	25
**Median age at diagnosis**	48
Years; range	36–77
**Median days between samples**	19
Days; range	6–42
**Tumour type**	
IDC	18 (72)
ILC	6 (24)
Other	1 (3)
**Tumour grade**	
1	6 (24)
2	13 (52)
3	5 (20)
Unknown	1 (4)
**Tumour size**	
≤ 10 mm	2 (8)
11–20 mm	8 (32)
21–50 mm	8 (32)
> 50 mm	6 (24)
Unknown	1 (4)
**Lymph node status**	
Positive	13 (52)
Negative	11 (44)
Unknown	1 (4)
**Lymphovascular invasion**	
Present	18 (72)
Absent	7 (28)

### Correlation in the 21-gene signature and experimental recurrence scores between paired breast cancer samples

Details of patient menopausal status or menstrual cycle stage at the time of tissue collection are not routinely reported, and this information was not available for this research. An arbitrary age of 50 is often used to define menopausal status in clinical studies [25]. However, as the perimenopausal period occurs over a highly variable time frame, lasts up to 10 years and is characterised by disrupted ovarian hormone secretion and irregular menses, the initial statistical analysis was conducted using age as a continuous variable.

**Fig. 3:**
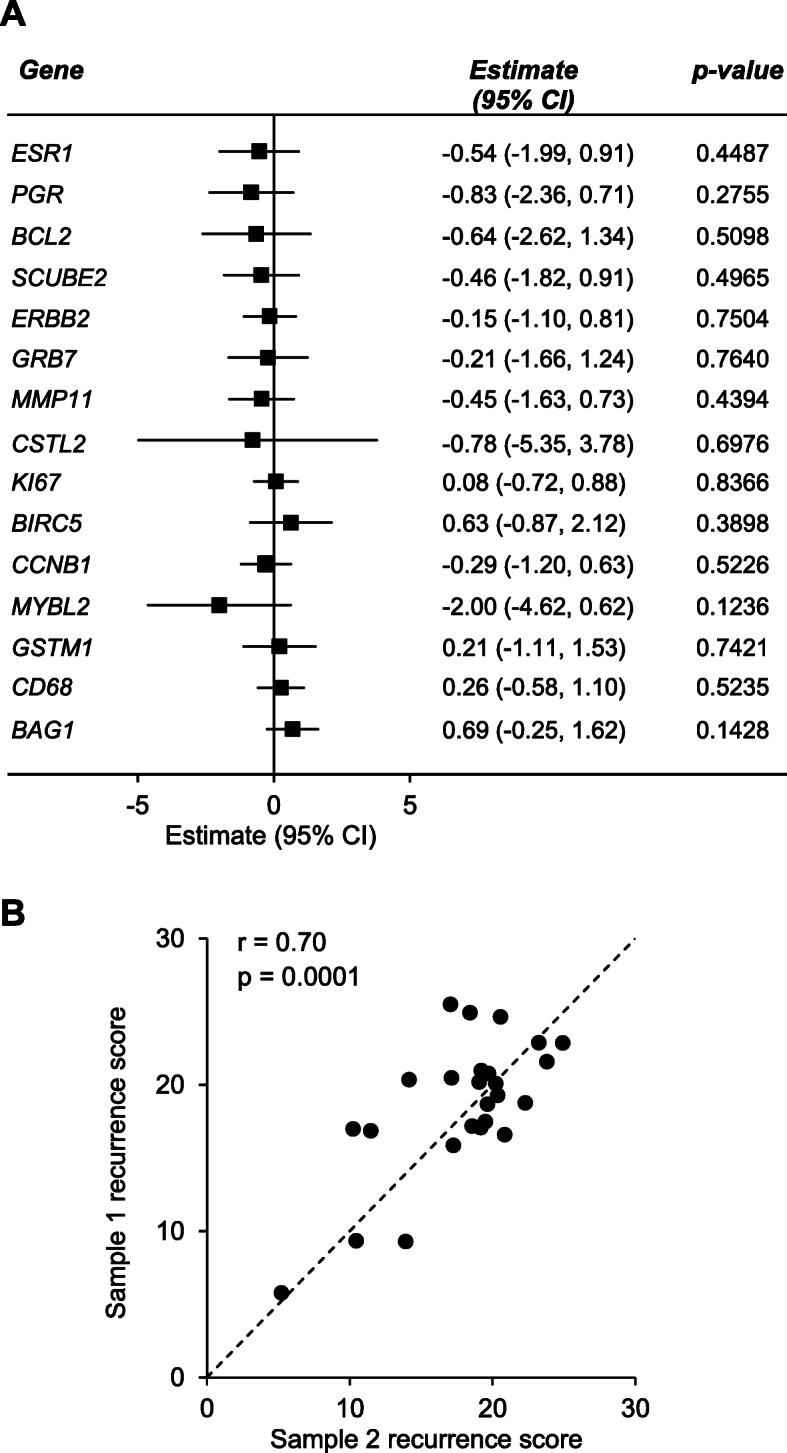
Agreement in the 21-gene signature and experimental recurrence scores between paired breast cancer samples. Paired breast cancer samples were collected from women with invasive, ER-positive breast cancer (*n* = 25). The 21-gene signature was assessed through real-time PCR. **a** Forest plot showing concordance in gene expression between sample 1 and sample 2, for the genes which comprise the Oncotype DX 21-gene signature. To determine if gene expression varied significantly between paired samples, statistical significance was assessed using linear mixed-effect models adjusted for multiple comparisons. No data were statistically significant (*p* > 0.05). **b** Correlation in experimental recurrence scores between paired samples. Recurrence scores were calculated from reference-normalised gene expression as described in the “Methods” section. Sample 1 corresponds to the first collected sample, presented against its corresponding later collected pair, sample 2. The dashed line represents perfect correlation, where deviation from the line reflects discordance between paired samples. Spearman’s correlations and *p* values are presented

Samples collected from the same breast cancer on different days are anticipated to show some variability in gene expression due to extrinsic factors such as the precise part of the tumour biopsied as well as differences in tumour fixation and processing [26, 27]. This variability is expected to be similar between premenopausal and postmenopausal women. Indeed, we found no significant differences between samples 1 and 2 in the expression of the 21 genes and patient age did not influence the magnitude of change in gene expression between paired samples when assessed using linear mixed-effect models adjusted for repeated measurements (*p* > 0.05; Fig. 3a).

From this reference-normalised gene expression data, a 21-gene experimental recurrence score was calculated for each sample. This recurrence score is analogous to the Oncotype DX Recurrence Score. The recurrence score of sample 1 significantly correlated with the recurrence score of sample 2 (Spearman’s correlation coefficient *r* = 0.70, *p* = 0.0001; Fig. 3b). Together, these findings confirm that variability in gene expression due to extrinsic factors was similar between premenopausal and postmenopausal women and that the two samples that comprise each pair are related in their gene expression signature.

### Experimental recurrence scores are more variable in paired samples collected from younger women and are driven by variable expression of Proliferation and HER2 group genes

**Fig. 4:**
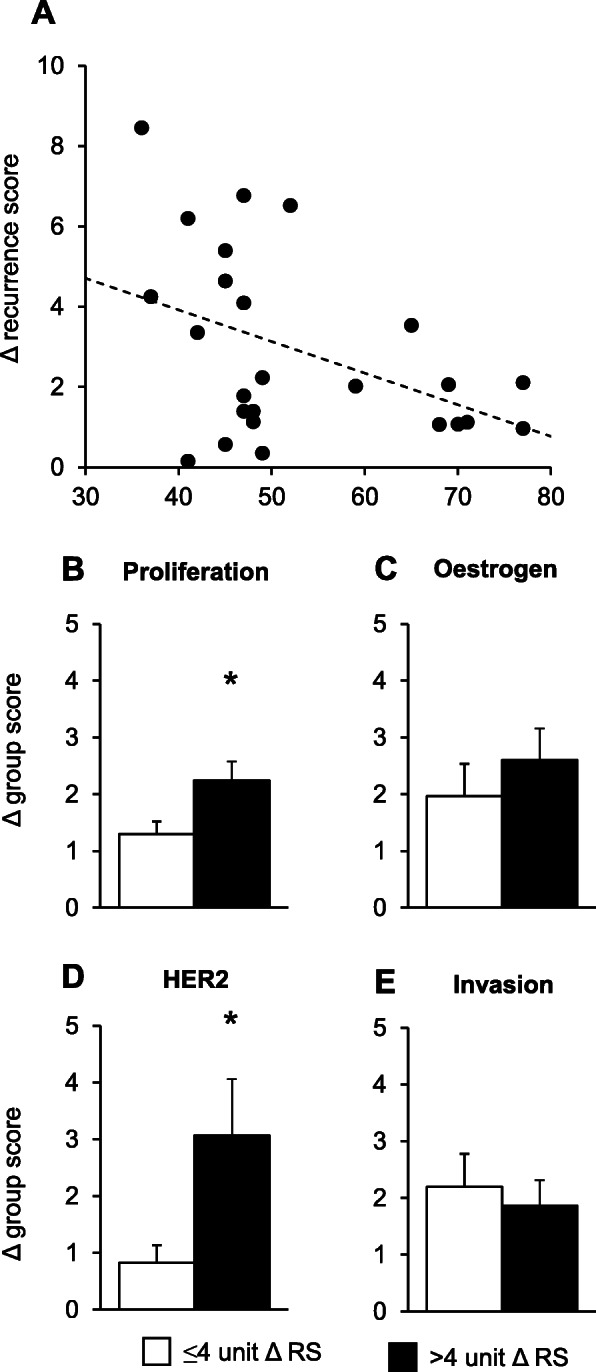
Variability in 21-gene experimental recurrence scores and 21-gene group scores between paired breast cancer samples. **a** Difference in 21-gene experimental recurrence scores (RS) between paired breast cancer samples by age. Recurrence scores were calculated from reference-normalised gene expression as described in the “Methods” section, and discordances were quantified by calculating the absolute difference in recurrence score between sample 1 and sample 2. Linear regressions were performed to investigate the association between the difference in recurrence score and age as a continuous variable. Data are presented as individual values. **b**–**e** Discordance in 21-gene group scores between paired breast cancer samples collected from younger women. For women aged < 50 years old, an arbitrary threshold of 4 units was set to distinguish between paired samples showing small differences in recurrence scores of ≤ 4 units (*n* = 9) and paired samples showing large differences in recurrence scores of > 4 units (*n* = 7). The 21-gene group scores were calculated for each tumour, as described in the “Methods” section, and changes in group scores between paired breast cancer samples were compared. The change in the **b** Proliferation group, **c** Oestrogen group, **d** HER2 group and **e** Invasion group scores between paired breast cancer samples. Results are presented as mean + SEM. Mean discordances were compared using the independent *t* test. Statistical significance was determined when *p* ≤ 0.05; an asterisk signifies *p* ≤ 0.05

To quantify discordances in recurrence scores between paired breast cancer samples, the absolute difference in recurrence score between sample 1 and sample 2 was calculated. Discordance was analysed with age as a continuous variable. There was a significant inverse association between patient age and discordance in the recurrence score. For every 1-year decrease in age, the difference in recurrence scores between paired samples increased by 0.08 units (95% confidence interval − 0.14, − 0.01; *p* = 0.017; Fig. 4a).

The underlying gene expression changes that contribute to the increased discordance in younger women were investigated. For patients under the age of 50 years, paired breast cancer samples with minimal discordances in recurrence scores were separated from paired samples with larger discordances by setting an arbitrary threshold of 4 units. The age of 50 was selected as it is the optimal age-based proxy to distinguish premenopausal women from postmenopausal women when menopausal status is unknown [25].

Paired breast cancer samples collected from younger women with discordance > 4 units showed greater differences in the expression of Proliferation (*p* = 0.04; Fig. 4b) and HER2 21-gene group scores (*p* = 0.03; Fig. 4d), compared to paired samples with discordance ≤ 4 units. The expression of Oestrogen (*p* = 0.44; Fig. 4c) and Invasion (*p* = 0.62; Fig. 4e) group scores did not differ significantly between groups. Patient and tumour characteristics were similar between groups, and not likely to contribute to discordance (Table 2). For women over the age of 50, only one sample showed a change in recurrence score greater than 4 units which could not be statistically analysed.

**Table 2:** Characteristics of patients aged < 50 years with discordances in recurrence scores ≤ 4 units, compared to > 4 units

Characteristics	≤ 4 unit change ***n*** (%)	> 4 unit change ***n*** (%)
**Total number**	9	7
**Median age at diagnosis**	47	45
Years; range	41–49	37–47
**Median days between samples**	23	14
Days; range	10–42	9–29
**Tumour type**		
IDC	8 (89)	5 (72)
ILC	1 (11)	1 (14)
Other	0 (0)	1 (14)
**Tumour grade**		
1	3 (33)	2 (29)
2	3 (33)	2 (29)
3	3 (33)	2 (29)
Unknown	0 (0)	1 (14)
**Tumour size**		
≤ 10 mm	2 (22)	0 (0)
11–20 mm	4 (44)	2 (29)
21–50 mm	2 (22)	2 (29)
> 50 mm	1 (11)	2 (29)
Unknown	0 (0)	1 (14)
**Lymph node status**		
Positive	2 (22)	3 (43)
Negative	7 (78)	3 (43)
Unknown	0 (0)	1 (14)
**Lymphovascular invasion**		
Present	2 (22)	1 (14)
Absent	7 (78)	6 (86)

## Discussion

The Oncotype DX 21-gene Recurrence Score assay is used to guide adjuvant chemotherapy treatment decisions for women with early-stage, ER-positive, HER2-negative breast cancer. However, the Recurrence Score algorithm was largely developed and validated for use in postmenopausal women, and whether menstrual cycling in premenopausal women affects the algorithm has been a remarkably underappreciated research question. Our analysis of paired breast cancer samples provides the first evidence that patient age affects concordance in 21-gene experimental recurrence scores between paired samples of the same tumour taken on different days. Discordance is primarily due to differences in the expression of genes associated with proliferation and HER2 signalling. Consequently, Recurrence Scores generated by the Oncotype DX algorithm may be critically affected by patient age and menstrual cycle stage.

As this work was conducted on archived breast cancers, there is no clinical information surrounding the patient’s menstrual histories or menopausal status. Identification of a direct effect of menstrual cycle stage and discordance in Recurrence Score in human breast cancers would require further large-scale prospective trials. However, age-related differences in chemotherapy benefit for women with intermediate Onctoype DX Recurrence Scores have already been demonstrated [10]. Incorporation of menstrual cycle stage into future prospective studies will assist in understanding this age-related difference in chemotherapy benefit.

Increased expression of Proliferation and HER2 group genes were largely responsible for the increased variability in recurrence scores observed between paired samples. The observed variability in younger women could be a factor of menstrual cycling, as previous studies report that tumour proliferation and HER2 gene expression fluctuate during the menstrual cycle, in accordance with concentrations of oestrogen and progesterone. In premenopausal women, the highest proliferative activity of breast epithelium [28] and breast cancer samples [29] is observed during the luteal phase, when circulating concentrations of progesterone peak. Additionally, in vitro and in vivo stimulation with oestrogen and/or progesterone promotes proliferation of breast cancer cells, an effect which can be reversed with anti-estrogenic treatment [30, 31]. The expression of HER2 also fluctuates across the menstrual cycle, with the highest expression during the luteal phase [32]. Likewise, progesterone treatment of breast cancer cell lines increases growth factor receptor signalling and Oncotype DX Recurrence Scores [24]. While it has also been reported that the expression of oestrogen-regulated genes fluctuates across the menstrual cycle [20–23] and that oestrogen and progesterone affect the invasive properties of premenopausal breast cancers [33–35], we did not observe variable expression of Oestrogen or Invasion group genes between paired breast cancer samples collected from younger women.

It is interesting to note that not all young women showed discordance in recurrence scores between paired breast cancer samples. As menstrual histories of women in this study were unknown, it is possible that tissue was collected at times of the cycle when concentrations of oestrogen and progesterone did not differ significantly, or the woman was in a perimenopausal state with anovulatory cycles. As such, the impact of menstrual cycle stage on recurrence score might only be seen when there is a large enough cycle-dependent difference in circulating hormone concentrations to impact gene expression. Furthermore, biological differences between tumours may also explain why discordances in recurrence scores were only observed in a subset of tumours. There is significant heterogeneity within ER-positive breast tumours that may influence responsiveness to ovarian hormones. Paired samples exhibiting large discordances in recurrence scores may be more sensitive to fluctuations in oestrogen and progesterone and more prone to cycle-induced changes. However, the underlying tumour biology that drives this increased susceptibility, and whether it is possible to identify tumours that are more sensitive to fluctuations in oestrogen and progesterone, warrants further investigation.

There is the potential to tailor gene expression-based assays for use in premenopausal women. Incorporation of menstrual cycle stage with the Oncotype DX algorithm could provide an opportunity to improve the accuracy of the predictive benefit of chemotherapy for premenopausal women with intermediate Recurrence Scores. Alternatively, refinement of the Oncotype DX algorithm through alteration of the weighting of Proliferation and HER2 group scores could form the basis of an improved assay for premenopausal women.

## Conclusions

Discordance in 21-gene experimental recurrence scores between paired breast cancer samples is inversely related to patient age and suggests that recurrence scores may be critically affected by the menstrual cycle stage at the time of tissue collection. Consequently, the use of the Oncotype DX algorithm to inform decision-making in premenopausal breast cancer patients could potentially lead to suboptimal or unnecessary treatments. Currently, there is a pressing need for consideration of patient age in the application of gene expression-based precision medicine for breast cancer.

## Data Availability

The datasets used and/or analysed during the current study are available from the corresponding author on reasonable request.

## Abbreviations

ACTB: Actin beta
BAG1: BCL2-associated athanogene
BCL2: B cell CLL/lymphoma 2
BIRC5: Baculoviral IAP repeat-containing 5 (survivin)
CCNB1: Cyclin B1
CD68: CD68 molecule
cDNA: Complementary DNA (cDNA)
CSTL2: Cathepsin L2
ER: Oestrogen receptor
ERBB2: Erb-B2 receptor tyrosine kinase 2
ESR1: Oestrogen receptor gene
FFPE: Formalin-fixed paraffin-embedded
GADPH: Glyceraldehyde-3-phosphate dehydrogenase
GRB7: Growth factor receptor-bound protein 7
GSTM1: Glutathione S-transferase mu 1
GUSB: Glucuronidase beta
HER2: Human epidermal growth factor receptor
MKI67: Marker of proliferation Ki-67
MMP11: Matrix metallopeptidase 11 (stromelysin 3)
MYBL2: Myb proto-oncogene like 2
PGR: Progesterone receptor gene
RPLP0: Ribosomal protein lateral stalk subunit P0
RS: 21-gene recurrence score
SCUBE2: Signal peptide, cub domain and Egf-like domain-containing 2
STK15: Aurora kinase A
TFRC: Transferrin receptor
